# Increased heart rate variability during evolving fetal hypoxia: Be careful during pushing efforts

**DOI:** 10.1111/aogs.14788

**Published:** 2024-01-24

**Authors:** Léa Descourvieres, Louise Ghesquiere, Charles Garabedian

**Affiliations:** ^1^ Department of Obstetrics Lille University Hospital Lille France; ^2^ University of Lille Lille Unit Research 2694‐METRICS Lille France

Sir,

We would like to thank Christopher Lear and his colleagues for their interest in our article.^1^ In our series, we had 42 cases with abnormal fetal heart rate (FHR) variability, including those with a saltatory FHR pattern and those with decreased variability.^2^ Among those, we had two cases with increased FHR variability during the second stage of labor.

The first case had a normal FHR pattern at the beginning of the labor. One hour before maternal active pushing, an increase in the baseline FHR was noted (from 150 to 170 bpm), with no decelerations and normal variability. There were no signs of intrauterine infection. The FHR variability increased with the start of expulsive effort, which lasted 13 minutes. Umbilical cord blood gas analysis showed arterial pH of 6.94 (venous pH was of 7.22), with a base deficit of 12.1 mmol/L and lactate of 8.8 mmol/L. At 1 hour 30 minutes after birth, the neonatal arterial pH was normal at 7.30.

In the second case, the FHR was initially normal, then decelerations appeared 70 minutes before active pushing. These were repeated and progressively deeper. At the start of expulsive efforts, a saltatory FHR pattern was present over 4 minutes, followed by bradycardia with decreased variability. Umbilical cord blood gas analysis showed arterial pH of 6.91 (venous pH was 7.15), with a base deficit of 13.5 mmol/L and lactate of 10.8 mmol/L. At 1 hour of life, the neonatal arterial pH remained low at 7.10.

In these two cases, we found that the saltatory FHR pattern appeared during maternal active pushing efforts after an abnormal FHR pattern had occurred before the expulsive efforts. This corroborates with the results of Loussert et al.^3^ In their prospective bicentric study, among the 4394 women included, 177 (4%) had a saltatory FHR pattern during the last 60 minutes before delivery. In a multivariable analysis, they showed that the marked variability was significantly associated with neonatal acidosis (adjusted relative risk 2.30, 95% confidence interval 1.53–3.44).

Thus, even though the majority of abnormalities in FHR variability are a reduced variability during labor, a fetus with saltatory FHR pattern, particularly during maternal expulsive efforts, must be considered at increased risk for hypoxia, which should be taken into account in the management of labor and delivery.
